# [Corrigendum] Changes in the interstitial cells of Cajal in the gallbladder of guinea pigs fed a lithogenic diet

**DOI:** 10.3892/etm.2025.13006

**Published:** 2025-10-29

**Authors:** Lei Huang, Chao Ding, Xinmin Si

Exp Ther Med 22:823, 2021; DOI: 10.3892/etm.2021.10255

Following the publication of the above article, the authors contacted the Editorial Office to explain that they made an error in the assembly of Fig. 4B, as shown on p. 6, which went uncorrected before the paper went to press. Essentially, the authors identified that non-representative images for Fig. 4B (the GAPDH control blots) were used during the preparation of the manuscript, which was uncovered during a thorough recheck of experimental data.

The revised version of Fig. 4, now showing the correct GAPDH control data in Fig. 4B, is shown below. Note that the error made in assembling this figure did not have an impact on either the results or the conclusions reported in the paper. All the authors agree with the publication of this corrigendum, and are grateful to the Editor of *Experimental and Therapeutic Medicine* for allowing them the opportunity to publish this; furthermore, they apologize to the readership for any inconvenience caused.

## Figures and Tables

**Figure 4:**
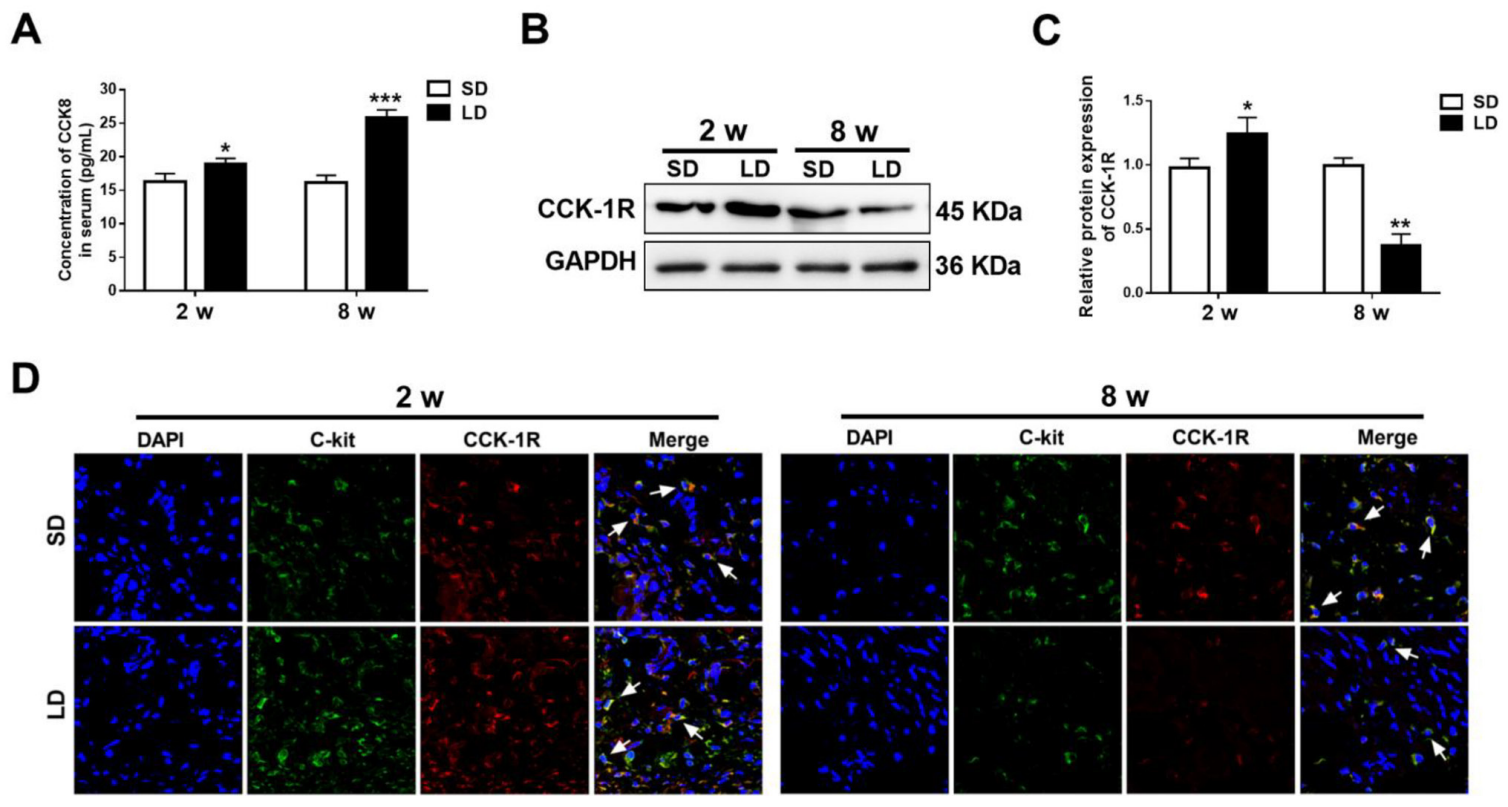
CCK1R expression in gallbladder ICCs is altered in guinea pigs fed a LD. (A) Serum CCK8 levels were measured after 2 and 8 weeks in guinea pigs fed a SD or LD. (B and C) Western blotting for CCK1R protein expression in guinea pig gallbladders fed a SD or LD. (D) Immunoﬂuorescence labelling of CCK1R (red) expression in C-kit (green)-positive gallbladder ICCs of guinea pigs fed a SD or LD. Magnification, x400. Cell nuclei were labelled using DAPI (blue). White arrowheads indicate C-kit-positive gallbladder ICCs. ^*^P<0.05, ^**^P<0.01 and ^***^P<0.001 vs. SD/2 weeks or SD/8 weeks. SD, standard diet; LD, lithogenic diet; ICCs, interstitial cells of Cajal; CCK1R, cholecystokinin receptor type A.

